# The Impact of Early Neighborhood Cohesion, and Its Mechanism, on Cognitive Function in Later Life

**DOI:** 10.3389/fpsyt.2022.848911

**Published:** 2022-04-27

**Authors:** Tao Zhou, Xiaoyi Zhang, Shuming Fan, Zeming Deng, Can Jiao

**Affiliations:** ^1^School of Psychology Shenzhen University, Shenzhen, China; ^2^Faculty of Education, Department of Educational Psychology, East China Normal University, Shanghai, China; ^3^The Shenzhen Humanities & Social Sciences Key Research Bases of the Center for Mental Health, Shenzhen University, Shenzhen, China

**Keywords:** cognitive function, early neighborhood cohesion, life course perspective, early friendship, depression, social activities engagement

## Abstract

**Objectives:**

This study aimed to explore the impact of early neighborhood cohesion, and its mechanism, on cognitive function in later life.

**Methods:**

In total, 10,727 Chinese elderly, aged 60–90, forming two datasets (2014 and 2018) from the China Health and Retirement Longitudinal Study (CHARLS) were used as a sample. Childhood neighborhood cohesion was measured by the extent of how much neighbors were willing to help and how close-knit neighbors were. Mini-Mental State Examination (MMSE) and Center for Epidemiologic Studies Depression Scale (CESD-10) were used to assess the cognitive functions and depression of the elderly. We used a structural equation model to examine the relationship between early neighborhood cohesion and late-life cognitive function and conducted bootstrapping analyses to assess the mechanism.

**Results:**

Cognitive function was positively predicted by childhood neighborhood cohesion (β = 0.06, *p* < 0.001), and cognitive function of the elderly were also positively predicted through the mediating effects of childhood friendships, which included depression and social activity participation as two chain paths.

**Conclusion:**

The findings suggest that childhood neighborhood cohesion positively predicts cognitive function among elderly people through the mediating roles of childhood friendship, depression, and social activity engagement. Childhood neighborhood cohesion is correlated with better childhood friendships, then to fewer depression symptoms and greater elderly social activity participation, and finally to better cognitive functions in the elderly.

## Introduction

China’s population is aging rapidly. Zhai et al. (1) predicted that the proportion of the elderly population aged 60 and above in China will reach 25% in 2030 and 37.92% in 2100. With an aging society, cognitive function among the older Chinese population becomes an important public health concern. Cognitive function refers to a spectrum of higher-order cerebral functions (2). It declines with age and can be sufficiently severe to threaten the physical and mental health of the elderly. Its decline is characterized as a clinical syndrome of dementia. The number of dementia patients in China is the largest in the world, placing a considerable strain on the public and health care services (3). As a major problem facing the elderly population, dementia may adversely affect the elderly’s ability to maintain social connections. Additionally, the elderly who have lost the ability to live independently place a heavy burden on their families and society. Cognitive decline is the most common cause of dementia (4). Therefore, it is necessary to prevent the cognitive decline of the elderly in China.

Researchers have proposed that early life factors related to cognitive aging should be identified in the context of the life course (5). The cognitive health of the elderly is not only affected by their current conditions but also the result of the accumulation of life experiences (6). Previous work has linked childhood experiences with cognitive function in later life. For example, older adults are at a lower risk of cognitive impairment if they had better childhood friendship experiences (7). Additionally, childhood family-life happiness significantly predicts older adult cognitive functioning. However, previous studies focused on the impact of early personal experiences on cognitive abilities in later life, but few studies have considered environmental factors in childhood. What deserves additional consideration is whether and how the childhood neighborhood social environment, such as neighborhood cohesion, impacts the cognitive function of the elderly. In addition, it is worth considering whether and how childhood cohesion affects the cognition function of the elderly.

Neighborhood cohesion is the degree of mutual trust, support, unity, and friendship among neighbors, which reflects the social environment of the neighborhood (8). Socialization theory posits that the early social environment integrates individuals into society, shaping their social characters, values, and behavioral patterns (9). Those who grew up in cohesive neighborhoods may show better social development (10), which impacts cognitive function in later life. On the basis of relevant literature regarding cohesive neighborhoods and cognitive function, we proposed the following hypothesis: Neighborhood cohesion in childhood is positively associated with cognitive function in the elderly.

Children who live in cohesive neighborhoods are likely to develop high-quality friendships. First, in cohesive neighborhoods, children are guided to take a pro-social attitude and conform to the norms of trust, solidarity, and reciprocity, which may contribute to the establishment of harmonious peer relationships (10). Second, according to the social learning theory (11), children can acquire social skills by observing and imitating how cohesive neighbors interact. These social skills can be applied to children’s interpersonal relationships, helping them form friendships (12). Finally, parents are willing to let their children play alone in common areas when they perceive their neighborhoods as cohesive (13). Those independently mobile trips provide great opportunities for children to make friends.

A recent study found that childhood friendship is significantly related to better cognitive function and slower cognitive decline in middle and old age (7). The cognitive reserve hypothesis postulates that participation in stimulating activities early in life can help the brain build a reserve to buffer against cognitive decline (14). It has been reported that a higher level of availability of cognitive resources and frequency of participation in cognitively stimulating activities at 12 years of age is associated with less cognitive decline per year, and the pathological symptoms of Alzheimer’s disease were mediated by these factors (15). As an important source of cognitive stimulation, early friendship experiences contribute to improving cognitive reserves, especially when educational resources are lacking. Childhood neighborhood cohesion may protect the cognitive function of the elderly by promoting childhood friendship. Therefore, we proposed the following second hypothesis: Childhood friendship mediates between childhood neighborhood cohesion and cognitive function in older adults.

Childhood neighborhood cohesion and friendship may also reduce depressive symptoms in old age, thereby improving cognitive function. Evidence is emerging that childhood friendship can protect elderly people from depression. The higher the quality of childhood friendship, the fewer depressive symptoms in adults (16). The elevated level of loneliness and decreased engagement in peer relationships during childhood are associated with increased depressive symptoms in middle and old age (9). Thus, depressive symptoms in later life are influenced by childhood neighborhood cohesion and friendship.

Other quantitative studies have demonstrated that depressive symptoms in later life confer a greater risk of cognitive decline (17–19). One study has shown that depressive symptoms in old age are negatively correlated with cognitive abilities, such as orientation, language, memory, attention, and calculation skills (19). The elderly people with more depressive symptoms performed worse on the cognitive tests, including delayed word recall, digit symbol substitution, and animal fluency (17). Longitudinal studies have also shown that depressive symptoms in the elderly negatively predict cognitive function (18). The feeling of depression distracts individuals’ attention and interferes with cognitive task performances (20). Elevated levels of inflammatory factors in depressed older adults may cause nerve damage and enlarge neuropathological changes related to Alzheimer’s disease (18). In conclusion, childhood friendship could independently mediate the relationship between childhood neighborhood cohesion and cognitive function in the elderly, and depressive symptoms could independently mediate the association of childhood friendship with cognitive function in later life. However, how the two mediators can work together in this relationship remains to be explored. According to Hayes (21), when there is more than one mediator in a mediation model, these mediators have chain-mediating roles if they are related to each other. Considering the effect of childhood friendship on depression, this study further proposed the following third hypothesis: Childhood friendship and depression play chain mediating roles between childhood neighborhood cohesion and cognitive function among the elderly.

Childhood neighborhood cohesion and friendship may also affect cognitive function by enhancing social activity engagement among older adults. Children’s success in fostering harmonious peer relationships increases friendship-related self-efficacy, which is the belief in the ability to communicate and interact with friends, resolve conflicts, and manage interpersonal emotions (16). They will be motivated to participate actively in social interactions and form a self-reinforcing cycle. On the contrary, poor peer relationships usually result in low-quality socialization, limiting social participation over a lifetime (22). The neurobiology literature shows that early social isolation has deleterious effects on brain functions related to the social functioning in adulthood, revealing a mechanism underlying sociability deficits (23). Moreover, children who lack friendship in childhood have limited opportunities to practice social skills, which restricts their social interactions as adults (24).

Based on the points mentioned above, childhood neighborhood cohesion affects social activity engagement by influencing childhood friendships. Additionally, social activity engagement is protective of cognitive function in elder adults (25). Participation in at least one social activity and the number of participated social activities in old age is significantly related to better mental status and memory (26). A study using a latent class growth analysis found that older people with active social activity engagement are more likely to have stable cognitive decline trajectories (27). The “Use It or Lose It” hypothesis postulates that social activity engagement provides the elderly with intellectual stimulation, helping them exercise brain function (28). This kind of cognitively stimulating lifestyle enables the elderly to better maintain their cognitive abilities (29). Therefore, we proposed a fourth hypothesis: Childhood friendship and social activity engagement play chain mediating roles between childhood neighborhood cohesion and cognitive function in later life.

**FIGURE 1:**
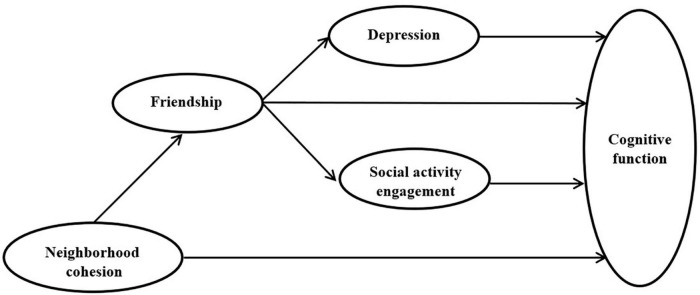
Hypothesized structural equation model (SEM).

In summary, the current study aimed to expand the “life course” model of later-life cognitive function by examining the relationship between childhood neighborhood cohesion and cognitive function in the elderly, as well as the mediating effects of childhood friendship, depression, and social activity engagement (Figure 1).

## Materials and Methods

### Data Source

This study analyzed data from the CHARLS,^1^ a nationally representative longitudinal survey of individuals over age 45 in China. The survey collects answers to a rich set of questions about aging, including physical and psychological health. Its samples were obtained using a multistage probability-proportional-to-size method in 150 counties across 28 provinces in China (30). In this study, the data on childhood neighborhood cohesion and friendship were taken from the CHARLS life course survey (2014). The data on late-life depression, social activity engagement, and cognitive function were obtained from the latest CHARLS follow-up survey (2018). We matched the individuals in CHARLS 2014 and 2018 using their IDs to link childhood experience with later life. When considering whether the social activity engagement of the most elderly is greatly affected by age, we selected subjects aged 60–90 years old. We used a maximum-likelihood estimation of missing data to maximize the utility of the available data (31). After excluding 17 samples with missing data for all variables, the final study sample included 10,727 respondents.

### Measurements

#### Childhood Neighborhood Cohesion

Childhood neighborhood cohesion was evaluated based on two aspects of the neighborhood in which respondents lived in before 17 years of age, as done previously (32). They were represented by the two following questions: (1) “Were the neighbors willing to help each other out?” (1 = not willing at all, 2 = not very willing, 3 = somewhat willing, and 4 = very willing); (2) “Were the neighbors close-knit?” (1 = not close-knit at all, 2 = not very close-knit, 3 = somewhat close-knit, and 4 = very close-knit).

#### Childhood Friendship

Childhood friendship was measured by the respondents’ experiences before age 17 in accordance with previous studies (7, 16, 33). This was determined using the following three questions: (1) “How often did you feel lonely because you did not have any friends?” (1 = often, 2 = sometimes, 3 = not very often, and 4 = never); (2) “Did you often have a group of friends that you felt comfortable spending time with?” (1 = never, 2 = not very often, 3 = sometimes, and 4 = often); (3) “Did you have a good friend?” (1 = no, 2 = yes).

#### Depression

Depression was assessed using the 10-item Center for Epidemiologic Studies Depression Scale (CESD-10) short form, which has been shown to have high reliability and validity. It has been widely used among older Chinese adults (34). This scale is based on 10 items having answers that reflect how respondents have felt and behaved during the last week, including “bothered by things”, “can’t concentrate”, “feel depressed”, “hard to do anything”, “feel hopeful”, “feel fearful”, “poor sleep”, “feel happy”, “feel lonely” and “can’t go on living”. Responses to each item are rated using a 4-point scale (0 = Rarely or none of the time; 1 = Some or a little of the time; 3 = Occasionally or a moderate amount of the time; 4 = Most or all of the time). The final CESD-10 score is the sum of each item, ranging from 0 to 30, with a higher score reflecting a greater level of severity. A CESD-10 score ≥ 20 was classified as depression group, ≥10 as the depressive symptoms group, and <10 as the no depressive symptoms group (19). The Cronbach’s α for CESD-10 was 0.807 in this study.

#### Social Activity Engagement

Measures of social activity engagement were assessed by whether the respondents had participated in the following six activities in the past month (1 = yes; 0 = no): (i) interacted with friends, (ii) played Mahjong, chess, or cards, or went to a community club; (iii) provided help to family, friends, or neighbors who does not live with the respondent; (iv) went to a sport, social, or other kind of club; (v) took part in a community-related organization; (vi) performed voluntary or charity work; and (vii) cared for a sick or disabled adult who does not live with the respondent.

#### Cognitive Function

Respondents’ cognitive function were measured using the Chinese version of the Mini Mental State Examination (MMSE), (2). This test uses 30 items to evaluate language (repeating phrase, three-step command, and naming), delayed memory, attention, calculation (calculation and copying intersecting polygons), immediate memory, and orientation (orientation to time and place). The total score ranges from 0 to 30 points, with higher scores indicating better cognitive function. The Cronbach’s α for MMSE was 0.686 in this study. The Chinese version of the MMSE shows adequate reliability and validity among patients with depression and in the general population (35).

#### Covariates

Based on previously published studies (7, 36, 37), the confounding variables in our study included sex (male or female), age (years), marital status (married or unmarried), and reading ability (illiterate and literate). In the regression model, we controlled for these confounding variables.

### Statistical Analyses

All statistical analyses were conducted using the SPSS 25.0 (SPSS Inc., Chicago, IL, United States). Results were considered statistically significant if *p* value was less than 0.05. The descriptive characteristics of the study sample were expressed as the means, standard deviation (SD), or proportion. Next, we used Structural Equation Modeling to explore the relationship and its mechanism between childhood neighborhood cohesion and cognitive function in the elderly using MPlus 7.11 (38). Given that some variables were categorical, such as childhood neighborhood cohesion and friendship, the model was assessed by the weighted least squares means and variance adjusted estimation. We used the following indices to assess the fit of the structural equation models: root means square error of approximation (RMSEA) < 0.08, comparative fit index (CFI) > 0.90, and Tucker-Lewis index (TLI) > 0.90 (39). The chi-square test (χ^2^) could have been reported as a fitness index, but it was excluded its use is not optimal for a large sample size. We handled the missing data using the pairwise present approach to maximize the utility of the available data. Finally, the mediation effects were estimated with 50,000 bootstrap samples.

## Results

Study sample characteristics are presented in Table 1. The mean age of participants were 69 years old, and females accounted for 50.9%, 68.4% of participants were literate, and 78.8% of participants were having companions. Most of the respondents lived in a neighborhood where people were close-knit (very close-knit, 42.0%, and somewhat close-knit, 52.6%) and willing to help each other (very willing, 45.0%, and somewhat willing, 40.1%). The majority of respondents never felt lonely because they did not have friends (77.5%), often had a group of friends (58.9%), and had a good friend (56.1%). About a third of the subjects had interacted with friends in the last month. The average cognitive function score was 21.15 (SD = 5.4), and the mean depression score was 8.42 (SD = 6.39).

**TABLE 1:** Descriptive characteristics.

Variable	*x* ± *SD/n* (%)
Age	69.09 ± 6.87
Female (%)	5460(50.9%)
Have companion (%)	8453(78.8%)
Non-illiterate (%)	7059(68.4%)
**Childhood neighborhood cohesion (%)**	
**Neighbors were willing to help**	
Very	2012(45.2%)
Somewhat	1773(39.8%)
Not very	435(9.8%)
Not at all	231(5.2%)
**Neighbors were close-nit**	
Very	1874(41.7%)
Somewhat	2362(52.6%)
Not very	192(4.3%)
Not at all	63(1.4%)
**Childhood friendship (%)**	
**Lonely due to no friends**	
Never	3475(77.5%)
Not very often	379(8.5%)
Sometimes	280(6.2%)
Often	348(7.8%)
**Having a group of friends**	
Often	2648(58.9%)
Sometimes	553(12.3%)
Not very often	451(10.0%)
Never	846(18.8%)
Having a good friend	2527(56.1%)
**Social activity engagement (%)**	
Interacted with friends	1626(30.4%)
Played Ma-jong, chess or cards, or went to community club	903(16.9%)
Provided help to family, friends, or neighbors who do not live with you	535(10.0%)
Went to a sport, social, or other kind of club	299(5.6%)
Took part in a community-related organization	119(2.2%)
Done voluntary or charity work	46(0.9%)
Cared for a sick or disabled adult	101(2.0%)
Depression	8.42 ± 6.39
Cognitive function	21.15 ± 5.44

*SD, standard deviation. Sample N = 10,727.*

**FIGURE 2:**
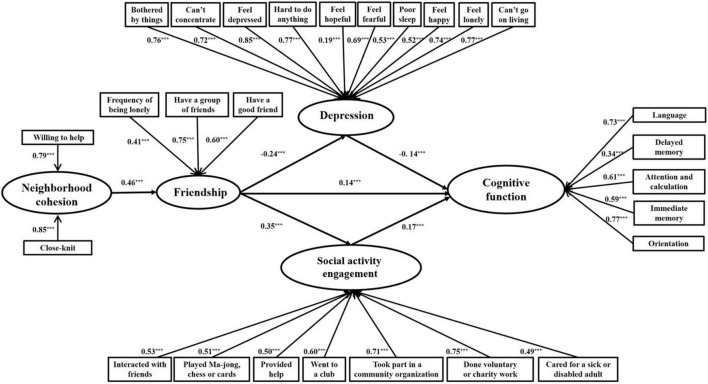
Final model with standardized path coefficients and significant level (*N* = 10,727). ***p < 0.001.

The cognitive scores for different categories of each explanatory variable and the statistical significance of the difference are shown in Table 2. There were significant differences in cognitive function among different categories of explanatory variables except for some social activities, such as “Interacted with friends” and “Cared for a sick or disabled adult.” The analysis of the measuring model indicated that the model had the appropriate factor structure. The factor loading from the latent variables to observed variables was significant, and the fit indices indicated the model fit the data well (RMSEA = 0.04, CFI = 0.96, and TLI = 0.95). As shown in Table 3, if other variables were not considered, the regression coefficient of childhood cohesion on cognitive function was significant (β = 0.16, *p* < 0.001) with good model fit indices (RMSEA = 0.02, CFI = 0.99, and TLI = 0.99). However, after entering childhood friendship, depression, and social activity engagement into the hypothesized Structural Equation Modeling, the path was not significant and had been deleted. The final model had good fit indices (RMSEA = 0.03, CFI = 0.94, and TLI = 0.93). Within the model, childhood neighborhood cohesion was positively associated with childhood friendship (β = 0.46, *p* < 0.001). Better childhood friendship predicted higher late-life cognitive function (β = 0.14, *p* < 0.001). Childhood friendship negatively predicted depression (β = -0.24, *p* < 0.001), which further negatively predicted cognitive function (β = -0.14, *p* < 0.001). Social activity engagement was also positively predicted by childhood friendship (β = 0.35, *p* < 0.001), and in turn, predicted higher cognitive function (β = 0.17, *p* < 0.001). The final model with standardized path coefficients and significance level are shown in Figure 2.

**TABLE 2:** Test for statistical differences in cognitive function scores.

Variable	Cognitive Function (*M* ± SD)	*P*
**Childhood neighborhood cohesion**		
Neighbors were willing to help		<0.001
Very	20.83 ± 5.36	
Somewhat	20.87 ± 5.34	
Not very	18.68 ± 5.42	
Not at all	17.50 ± 5.43	
Neighbors were close-nit		<0.001
Very	20.77 ± 5.42	
Somewhat	20.55 ± 5.38	
Not very	18.31 ± 5.54	
Not at all	16.36 ± 5.29	
**Childhood friendship**		
Lonely due to no friends		<0.001
Never	18.73 ± 5.67	
Not very often	20.09 ± 5.16	
Sometimes	19.40 ± 5.27	
Often	20.83 ± 5.39	
Having a group of friends		<0.001
Often	21.23 ± 5.21	
Sometimes	20.91 ± 5.25	
Not very often	19.43 ± 5.10	
Never	17.90 ± 5.78	
Having a good friend		<0.001
Yes	21.19 ± 5.24	
No	19.94 ± 5.51	
**Social activity engagement**		
Interacted with friends		0.635
Yes	21.17 ± 5.47	
No	20.31 ± 5.38	
Played Mahjong, chess or cards, or went to community club		<0.001
Yes	22.29 ± 4.60	
No	20.17 ± 5.52	
Provided help to family, friends, or neighbors who do not live with you		0.068
Yes	21.30 ± 5.20	
No	20.48 ± 5.44	
Went to a sport, social, or other kind of club		<0.001
Yes	22.26 ± 4.71	
No	20.47 ± 5.45	
Took part in a community-related organization		0.008
Yes	22.88 ± 4.64	
No	20.52 ± 5.43	
Done voluntary or charity work		0.020
Yes	23.16 ± 4.65	
No	20.54 ± 5.42	
Cared for a sick or disabled adult		0.738
Yes	21.20 ± 5.30	
No	20.56 ± 5.43	
**Depression**		<0.001
No depression	21.93 ± 4.97	
Depressive symptoms	19.80 ± 5.12	
Depression	18.18 ± 5.25	

*SD, standard deviation.*

**TABLE 3:** Path coefficient test

Path	β	95% CI	
		Low	High
Childhood neighborhood cohesion → cognitive function	0.06***	0.03	0.10
Childhood neighborhood cohesion → childhood friendship	0.46***	0.42	0.49
childhood friendship → cognitive function	0.14***	0.09	0.18
childhood friendship → depression	−0.24***	−0.27	−0.2
Depression → cognitive function	−0.14***	−0.17	−0.12
childhood friendship → social activity engagement	0.35***	0.29	0.41
social activity engagement → cognitive function	0.17***	0.13	0.2

*CI, confidence interval. Confidence intervals that do not contain 0 are significant.*

***p < 0.001.

As shown in Table 4, the indirect effect on the childhood neighborhood cohesion → childhood friendship → cognitive function path was 0.06, and the 95% confidence interval (CI) was [0.04, 0.09]. The childhood neighborhood cohesion → childhood friendship → depression → cognitive function path was 0.02, and the 95% CI was [0.01, 0.02]. The childhood neighborhood cohesion → childhood friendship–social activities engagement → cognitive function path was 0.03, and the 95% CI was [0.02, 0.04]. The total mediating effect was 0.11, and the 95% CI was [0.09, 0.13]. Because the 95% confidence intervals of the mediating effects all included zero, the second, third, and fourth hypotheses were proven.

**TABLE 4:** The mediation effect of childhood neighborhood cohesion on cognitive function.

Effect	Path	Effect	95% CI	
			Low	High
Direct Effect	Childhood neighborhood cohesion → cognitive function	0.004	−0.04	0.03
Indirect Effect	Childhood neighborhood cohesion → childhood friendship → cognitive function	0.06***	0.04	0.09
	Childhood neighborhood cohesion → childhood friendship → depression→cognitive function	0.02***	0.01	0.02
	Childhood neighborhood cohesion → childhood friendship → social activity engagement→cognitive function	0.03***	0.02	0.04
Total Mediating Effect		0.11***	0.09	0.13

*CI, confidence interval. Confidence intervals that do not contain 0 are significant.*

***p < 0.001.

## Discussion

This study provided the evidence that childhood neighborhood cohesion had prospective associations with cognitive function in the elderly. We further expanded this relationship by examining it in the framework of life course, and we determined the mediating role of childhood friendship, depression, and social activity engagement through which childhood neighborhood cohesion affects cognitive function.

According to the ecological system theory proposed by Jørgensen (40), the environment affects the development of individuals. The individual’s environmental system consists of four systems, include the micro system, the intermediate system, the outer system, and the macro system. The micro-system is the direct environment for individual activities and social interactions, which is constantly changing and developing. As children grow, the range of activities expands into the community, and their behavior is more easily influenced by the community environment (40). Under the research framework of Life Course Theory, the individual’s early experiences have a great influence on their behavior, and many studies have shown that the influence of social structure in childhood is more prominent and homogenous than that in adulthood (41), which laid the foundation for our research. In recent years, researchers have begun to pay attention to the influence of the growth environment in the early years on the individual’s future life, such as early life stress has an impact on cognitive function in the middle and later stages of an individual. This study focused on the relationship between childhood neighborhood cohesion and cognitive function in the elderly in a community environment.

Childhood neighborhood cohesion positively predicts childhood friendship and thereby contributes to cognitive health. The cohesive neighborhood may result in strong friendships in early life. The neighborhood is an important part of the social environment from which children form their values. The pro-social values that comprise the cohesion can be passed to children, promoting friendship (10). Furthermore, as supported by social learning theory (11), cohesive neighbors may be good role models for children as they build friendly relationships. Children can imitate their social behavior to develop social skills, such as empathy, sharing, and trust, which are important for friendship (12). Individuals with a higher quality of childhood friendship have better cognitive function in later life. This is consistent with previous studies (15, 36) and in line with the cognitive reserve hypothesis (14). There are multiple pathways between childhood friendship and cognitive function in older adults, and previous studies have explored their relationship from different perspectives. Burr and his colleagues (7) found that childhood friendship has an impact on cognitive function in the elderly through adult social disconnectedness and loneliness. Sharifian discussed the relationships among childhood friendship, activity engagement, and cognitive function in older adults (42). According to the Convoy Theory by Antonucci et al. (43), friendship is related to cognition, feelings, and behaviors through the interactions of individual and social factors. Because depression is a personal factor related to childhood friendship and cognitive function, it is a common mental health problem faced by the elderly in China, and social activity engagement is an important form of social contact. Therefore, in our study, depression and social activity engagement were selected as mediating variables to explore the relationship between childhood friendship and cognitive function. Early social interactions may provide beneficial cognitive stimulation and enhance cognition reserve, which can promote the development of the brain and reduce the pathological changes related to Alzheimer’s disease in old age (15). In addition, friendship can enrich the individual’s early life activities, helping them maintain an active mind in the future (36).

Some studies have suggested that cognitive function plays a role in depression and that depression and cognitive disorders, like dementia, share some biological mechanisms (44), with depression being a symptom of cognitive impairment (45). However, there is a bidirectional relationship between depression and cognitive function, and most studies have shown that while depression can predict cognitive decline, changes in cognitive function cannot predict depression (45, 46). This study found that childhood neighborhood cohesion and friendship further affect the symptoms of depression in old age, thereby forming a link to cognitive function. Individuals growing up in a cohesive neighborhood are likely to establish good peer relationships. Researchers have shown that a life devoid of friendship may induce stress and lead to dysfunctions in stress-related response systems, such as increases in stress hormones and inflammatory factors. When encountering stressful life events as adults, those who lacked peer relationships in childhood are prone to maladaptation and depressive symptoms (6). These supportive relationships may help them strengthen self-resilience and build skills that help recognize and manage emotions, which can aid in stress management and the prevention of depression. Conversely, the lack of neighborhood cohesion can lead to poor friendship (9, 16). If these responses continue through life, then they may permanently change the stress system over time and cause the failure of the emotional regulation, which confers a greater risk of depression in later years (6). Previous studies have found that depression can increase the levels of inflammatory cytokines and cortisol to levels that damage the neural connections in the brain and impair cognitive function (18).

Our results also indicate that childhood neighborhood cohesion and friendship impact cognitive function through social activity engagement in old age. In a neighborhood with greater cohesion, individuals form better peer relationships, which lay the foundation for social activity participation in later life. This occurs because childhood friendship enhances the individuals’ self-efficacy in interpersonal interactions. When individuals believe in their ability to communicate with others and cope with conflicts, they are motivated to make friends and form a supportive circle (24). Individuals with good friendships have numerous opportunities to practice social skills, during which neural circuits related to social interactions are fully developed and function normally until old age (23). Here, social activity engagement was significantly related to cognitive function among older people, which is consistent with the “Use It or Lose It” hypothesis (28) and previous findings (26, 27, 29, 47). Social activities provide cognitive stimulation to exercise the brain and keep the mind active, which contributes to cognitive health.

A key strength of this research is the refinement of the social convoy theory, which is mainly used to study the social relations of the elderly. The theory proposes that individuals go through life with different sets of supportive relationships separated by circles in terms of closeness (48). Specifically, the close relationships in the inner cycle are stable and will continue to stay through the whole life, whereas the marginal relationships in the outer cycle are likely to change with age. The theory has two research focuses, the dynamic changes in the social relations of the elderly during aging and the emotional closeness in the social network. The emotional “depth” and emotional “breadth” attributes of individual emotional intimacy have important impacts on the distribution of social relationships and satisfaction with social support networks. The influencing factors of the convoy theory include personal and situational characteristics, and their interactions affect the individual’s social network, as well as their health and subjective well-being (43, 49, 50). This study found that the neighborhood cohesion of the elderly in childhood affects social participation in old age, which is a continuous and stable process. Social relationships in childhood may change as people age; however, the emotional intimacy of the social network in childhood will affect the social participation of the elderly, and this will continue to affect the elderly, even in their later years. Early high-quality peer interactions may help individuals build social skills and lay the foundation for later social engagement. The current study indicated that although the size and composition of the outer social network will change along with circumstances, the process of individuals integrating into the social environment and expanding social relationships has a certain degree of continuity and stability.

An additional strength of the research is the highlighting of the importance of social life in the framework of the life course. Social environment (neighborhood cohesion), social relationship (friendship), and social activity engagement throughout life were found to exert significant and long-lasting influences on emotional and cognitive health. From the perspective of evolutionary psychology, social life has evolved into a deep-rooted human characteristic, which acts as a crucial means for ensuring survival and reproduction (51). Thus, interventions to encourage an active social life over the life course are beneficial. Many studies have shown that participating in social activities in early life (7) and later life (26, 27, 29) improves cognitive abilities. However, this evidence was limited to the individual level. Considering that effective public health interventions must be carried out in a multi-level and systematic manner (52), the current study fills the research gap by proving the effect of neighborhood cohesion, a situational factor, on cognitive health. Thus, public health initiatives may promote an active social life among residents of all ages by facilitating cohesion, thereby contributing to cognitive health in an increasingly aging society.

This study has certain limitations. First, many other factors affect cognitive function over the life course. Second, the self-reported data on the childhood experience may have recall errors, which may bias results toward the null hypothesis. Third, personality traits, such as extroversion and openness, may affect the relationship between childhood friendship and social activity engagement in old age. Finally, the relationship between social activity engagement and cognitive function may be bidirectional. In addition, a cross-lagged analysis can be used to examine directional influences between social activity engagement and cognitive function over time. Future research will explore the interaction effect of neighborhood cohesion (external factor) and personality (internal factor) on social life. Because green spaces provide venues for human social activities, they may contribute to cognitive health by enhancing neighborhood cohesion, which can be studied in the future.

## Conclusion

This study found that childhood neighborhood cohesion positively predicts the cognitive function of the elderly through the mediation effect of childhood friendship. This mediating effect includes two paths: “childhood friendship → depression” and “childhood friendship → social activity engagement”. Childhood neighborhood cohesion is related to better childhood friendship, then to fewer depression symptoms and more social activity participation over a life span, and finally to better cognitive function in the elderly.

## Data Availability Statement

The original contributions presented in the study are included in the article/supplementary material, further inquiries can be directed to the corresponding author.

## Author Contributions

TZ: validation and investigation. XZ: writing—review and editing, and methodology. SF: conceptualization and formal analysis. ZD: software. CJ: supervision. All authors contributed to the article and approved the submitted version.

## Conflict of Interest

The authors declare that the research was conducted in the absence of any commercial or financial relationships that could be construed as a potential conflict of interest.

## Publisher’s Note

All claims expressed in this article are solely those of the authors and do not necessarily represent those of their affiliated organizations, or those of the publisher, the editors and the reviewers. Any product that may be evaluated in this article, or claim that may be made by its manufacturer, is not guaranteed or endorsed by the publisher.

## Footnotes

1
http://charls.pku.edu.cn/
